# Gut microbiome associations with breast cancer risk factors and tumor characteristics: a pilot study

**DOI:** 10.1007/s10549-020-05702-6

**Published:** 2020-05-28

**Authors:** Anna H. Wu, Chiuchen Tseng, Cheryl Vigen, Yang Yu, Wendy Cozen, Agustin A. Garcia, Darcy Spicer

**Affiliations:** 1 Department of Preventive Medicine, Keck School of Medicine, University of Southern California, 1441 Eastlake Avenue, Rm 4443, Los Angeles, CA 90089 USA; 2 Mrs. TH Chan Division of Occupational Science and Occupational Therapy, University of Southern California, Los Angeles, CA USA; 3 Global Data Science and Analytics- Medical Device Division, Abbott Laboratories, Santa Clara, CA USA; 4 Hematology Oncology, Louisiana State University School of Medicine, New Orleans, LA USA; 5 Department of Medicine, Keck School of Medicine, University of Southern California, Los Angeles, CA USA

**Keywords:** Microbiome, Tumor characteristics, HER2 status, Age at menarche

## Abstract

**Objective:**

To investigate the association between gut microbiome with breast tumor characteristics (receptor status, stage and grade) and known breast cancer risk factors.

**Methods:**

In a pilot cross-sectional study of 37 incident breast cancer patients, fecal samples collected prior to chemotherapy were analyzed by 16S ribosomal RNA (rRNA) gene-based sequencing protocol. Alpha diversity and specific taxa by tumor characteristics and breast cancer risk factors were tested by Wilcoxon rank sum test, and by differential abundance analysis, using a zero-inflated negative binomial regression model with adjustment for total counts, age and race/ethnicity.

**Results:**

There were no significant alpha diversity or phyla differences by estrogen/progesterone receptor status, tumor grade, stage, parity and body mass index. However, women with human epidermal growth factor receptor 2 positive (HER2+) (*n* = 12) compared to HER2− (*n* = 25) breast cancer showed 12–23% lower alpha diversity [number of species (OTU) *p* = 0.033, Shannon index *p* = 0.034], lower abundance of *Firmicutes* (*p* = 0.005) and higher abundance of *Bacteroidetes* (*p* = 0.089*).* Early menarche (ages ≤ 11) (*n* = 11) compared with later menarche (ages ≥ 12) (*n* = 26) was associated with lower OTU (*p* = 0.036), Chao1 index (*p* = 0.020) and lower abundance of *Firmicutes* (*p* = 0.048). High total body fat (TBF) (> 46%) (*n* = 12) compared to lower (≤ 46%) TBF was also associated with lower Chao 1 index (*p* = 0.011). There were other significant taxa abundance differences by HER2 status, menarche age, as well as other tumor and breast cancer risk factors.

**Conclusions and relevance:**

Further studies are needed to identify characteristics of the human microbiome and the interrelationships between breast cancer hormone receptor status and established breast cancer risk factors.

## Background

In the past decade numerous intriguing links between the gut microbiota and risk of obesity, metabolic diseases and inflammatory responses have been reported [1, 2] but less is known about the gut microbiota of breast cancer patients [3, 4]. A study conducted in Kaiser Permanente health care members of pretreatment samples showed that after adjusting for age, body mass index (BMI), and other factors, postmenopausal women diagnosed with incident breast cancer (*n* = 48) compared to control women (*n* = 48) showed significantly lower alpha diversity in fecal microbiota, and differing relative abundance of select taxa of *Firmicutes* (*Clostridiaceae, Faecalibacterium, Ruminococcaceae, Dorea and Lachnospiraceae)* [5]. Low gut microbial diversity has been associated with obesity, insulin resistance, and other factors some of which are aligned to risk of breast cancer [6]. In a case-only study of 31 women diagnosed with early stage breast cancer [7], the total number of unique species of *Bacteroidetes,* and *Firmicutes* differed significantly by tumor stage and abundance of *Firmicutes* was 16% lower among those with overweight BMI (≥ 25 kg/m^2^) than those with normal BMI (*p* = 0.06).

Breast cancer is a heterogenous disease with multiple subtypes that display distinct risk factor patterns with differences between estrogen receptor (ER)/progesterone receptor (PR) positive (ER+PR+) versus those that are negative for ER/PR [8–10]. Breast cancers that are positive for human epidermal growth factor (HER2+) also differ from those that are HER2−, and triple negative (ER−PR−HER2−) breast cancers are the most deadly [9, 11]. It is not known whether different breast cancer subtypes are associated with distinct microbial signatures. Several studies have also explored the role of breast tissue microbiome in modulating the risk of breast cancer [12–17]. We are aware of one study that applied a pan-pathogen microarray (PathoChip) strategy on formalin fixed paraffin embedded samples of breast tissues to investigate microbial patterns by different breast cancer subtypes, but this study lacked information on tumor stage or grade or breast cancer risk factors [18].

We describe below results from a cross-sectional analysis conducted among 37 women diagnosed with incident breast cancer in Los Angeles County to further investigate whether gut microbiome prior to breast cancer chemotherapy differs by receptor status (ER, PR, HER2) and stage and grade of breast cancer. We also investigated whether gut microbiome profile differed by well-established breast cancer risk factors including age at menarche, parity, baseline BMI, and physical activity.

## Materials and methods

### Patient population and specimen collection

**Fig. 1:**
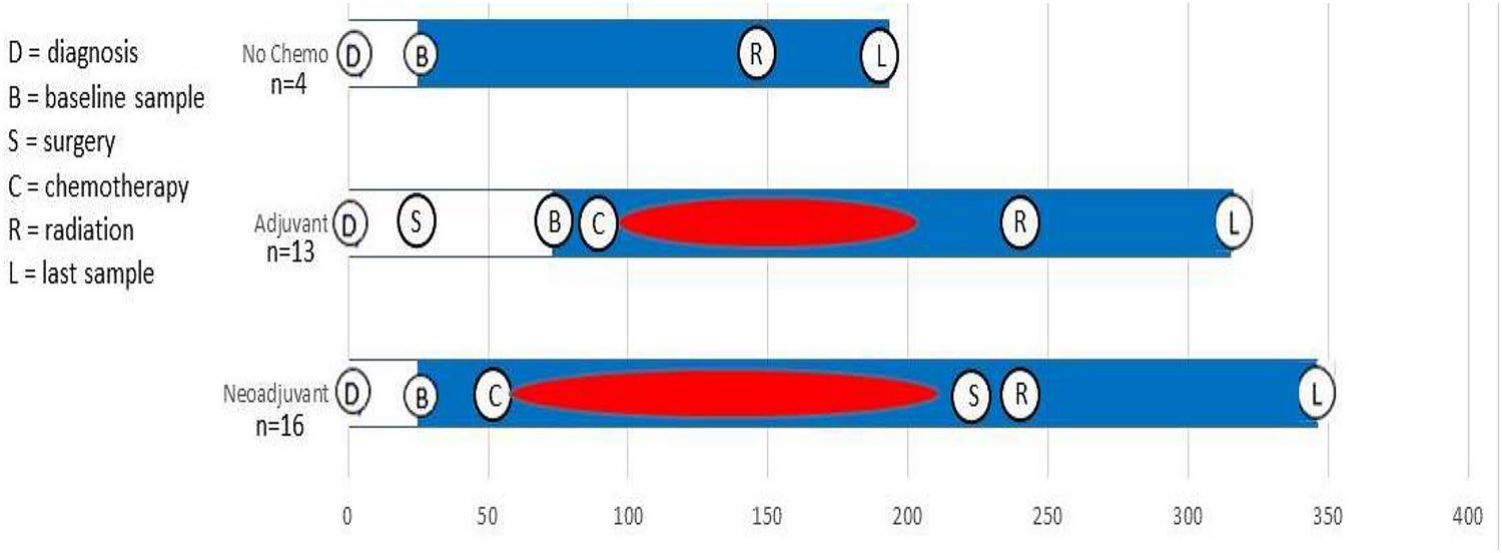
Collection of baseline (B) and last (L) fecal samples from study participants

This study was conducted at the University of Southern California (USC) Norris Comprehensive Cancer Center and at the Los Angeles County + USC Medical Center. Women of all race/ethnicities, newly diagnosed with incident invasive breast cancer were considered potentially eligible. Exclusionary criteria included recurrent breast cancer, a history of other cancers (other than non-melanoma skin cancer), celiac disease, inflammatory bowel disease, bariatric surgery, pregnancy or nursing within past 12 months, past treatment with chemotherapy, antibiotic use (defined as 1 week or more during the month prior to baseline fecal sample collection), or use of probiotic supplements or prednisone. After signing informed consent, eligible and willing patients donated up to four fecal specimens and completed up to four clinical visits during an average of 9 months follow-up. Baseline specimens were collected before chemotherapy started for those who received neoadjuvant chemotherapy and were collected after surgery but before chemotherapy for those who received adjuvant chemotherapy or only had surgery (Fig. 1). The study protocol was approved by the USC Institutional Review Board.

We used a fecal specimen collection kit with illustrated instructions that was designed and tested at the University of Maryland [19]. Participants were given collection kits and obtained samples using the provided pre-labeled collection devices and tubes containing the nucleic acid preservative RNAlater. All fecal samples were discreetly stored in the participants’ home freezers, and were either picked up by the study staff or brought in to USC by the study participants. These stool samples were then stored in the – 80 °C freezers of Preventive Medicine laboratory at USC until they were sent for measurement at the completion of the study. Body composition data obtained from the dual-energy x-ray absorptiometry (DEXA) scans at the first clinic visit (baseline) were included in our analysis. The DEXA scan was conducted at the USC Integrative Center for Oncology Research in Exercise. Participants also completed a baseline questionnaire to assess menstrual and reproductive history, medical history (e.g., hypertension, diabetes, benign breast diseases), family history of cancer, use of medications, and other lifestyle factors. Only the baseline fecal sample, i.e., collected before chemotherapy was included in the data analysis of this paper. Fecal samples collected during and after completion of breast cancer treatment are still under investigation.

### Fecal specimen processing and microbiome analyses

Microbiome analyses were conducted in the laboratory of Dr. Jacques Ravel using his well-established methods, including DNA extraction, 16S rRNA gene amplification of the two barcoded universal primers 319F and 806R for PCR amplification of the V3 and V4 hypervariable regions and sequencing the amplicons on the Illumina MiSeq platform [5, 19]. The 16S rRNA genes were amplified in 96-well microtiter plates. Negative controls without a template were processed for each primer pair. They performed taxonomic assignments and generated taxa abundance and read count tables for each of the 144 fecal samples we collected from 38 breast cancer patients. After we excluded 14 samples with low (< 100) read counts (referred to as failed), 130 samples remained from 37 patients as all 4 samples failed in one patient and she was excluded from all subsequent analyses. Hence this current analysis is comprised of baseline samples from 37 women diagnosed with incident breast cancer (Table 1).

**Table 1:** Characteristics of 37 breast cancer patients by human epidermal growth factor receptor 2 (HER2) status [*N *(%) or M ± SD]

	All	HER2 status		*p* value
		Negative	Positive	
*N*	37	25	12	
Mean age ± SD	50.6 ± 12.3	51.7 ± 13.7	48.3 ± 8.93	0.43^a^
Menopausal status				
Premenopause	20 (54)	12 (48)	8 (67)	
Postmenopause	17 (46)	13 (52)	4 (33)	0.32^b^
Race/ethnicity				
Hispanic	27 (73)	18 (72)	9 (75)	
Non-Hispanic	10 (27)	7 (28)	3 (25)	1.00^b^
Body mass index (BMI), kg/m^2^				
Mean BMI ± SD	30.6 ± 7.9	31.2 ± 8.3	29.5 ± 7.1	0.67^a^
< 25	9 (24)	7 (28)	2 (17)	
25–30	14 (38)	8 (32)	6 (50)	
> 30	14 (38)	10(40)	4 (33)	0.67 ^b^
Total body fat (TBF)				
Mean % fat ± SD	42.7 ± 6.9	42.6 ± 7.5	42.9 ± 5.8	0.90^a^
≤ 46%	25 (68)	19 (76)	6 (50)	
> 46%	12 (32)	6 (24)	6 (50)	0.15^b^
BMI and TBF				
I (< 25 & ≤ 46%)	9 (24)	7 (28)	2 (17)	
II (≥ 25 & ≤ 46%)	16 (43)	12 (48)	4 (33)	
III (≥ 25 & > 46%)	12 (32)	6 (24)	6 (50)	0.36^b^
Age at menarche				
Mean age ± SD	12.4 ± 1.5	12.2 ± 1.5	12.7 ± 1.5	0.49^a^
≤ 11	11 (30)	7 (28)	4 (33)	
12	9 (24)	7 (28)	2 (17)	
≥ 13	17 (46)	11(44)	6 (50)	0.81 ^b^
Parity				
Mean parity ± SD	1.8 ± 1.3	1.8 ± 1.2	1.9 ± 1.6	0.73^a^
No	8 (22)	6 (24)	2 (17)	
1–2	18 (49)	13 (52)	5 (42)	
≥ 3	11 (30)	6 (24)	5 (42)	0.65 ^b^
Stage at diagnosis				
I/II	22 (59)	15 (60)	7 (58)	
III	15 (41)	10 (40)	5 (42)	1.00^b^
Grade of tumor				
I/II	14 (38)	12 (48)	2 (17)	0.08^b^
III	23 (62)	13 (52)	10(83)	
ER/PR status				
ER+PR+	23 (62)	19 (76)	4 (33)	
ER+PR−	5 (14)	0 (0)	5 (42)	
ER−PR−	9 (24)	6 (24)	3 (25)	0.001^b^

^a^Wilcoxon rank sum test between HER2+ vs HER2− group for age, BMI, parity, and age at menarche

^b^Fisher exact test between HER2+ vs HER2− group for all other variables

### Statistical analyses

Microbiome alpha diversity was estimated after rarefaction using four measures: (a) counts of observed species (OTUs) unadjusted for relative abundances; (b) Chao1 as an estimate of the species richness; (c) Shannon index to measure both richness and evenness, and (d) phylogenetic distance (PD whole tree) in the diversity calculation. We used Wilcoxon rank sum test to examine differences in the alpha diversity between any two groups of interest (e.g., HER2+  vs HER2−) and Kruskal–Wallis to examine differences between any three groups of interest (e.g., age at menarche ≤ 11, 12, ≥ 13).

We conducted permutational multivariate analysis of variance (PERMANOVA) to test statistical significance of overall composition and to examine the relationship with personal factors including age (< 50, 50+), race (Hispanic, not Hispanic), menopausal status (pre- menopause, post-menopause); age at menarche (≤ 11, ≥ 12), BMI(< 25, ≥ 25), total body fat (TBF)(≤ 46%, > 46%), parity (nulliparous, parous), physical activity (no, yes), and tumor characteristics including stage(I/II, III), grade (I/II, III); receptor status (ER/PR: ER+PR+, ER+PR−, ER−PR−) and HER2 status (HER2−, HER2+).

The relationship of overall gut microbiome composition with personal factors (age, menopause status, race/ethnicity, age at menarche, parity, physical activity, BMI, TBF) and tumor characteristics was assessed by principal coordinate analysis (PCoA) based on the unweighted (qualitative) UniFrac distance matrix [20]. PCoA plots were generated using the first two principal coordinates, according to categories of personal and tumor characteristics.

Turning to taxonomy, we investigated the 201 specific genera that were present in at least 25% of our study samples. To accommodate the sparse, non-normally distributed count data, we conducted differential abundance analysis, using a zero-inflated negative binomial regression (NBR) model [21] provided by SAS proc genmod, to examine relationships of specific taxa to tumor characteristics and breast cancer risk factors. We investigated differences in taxa between groups with adjustment for total counts (Model 1), as well as age (< 49, 50–59, 60+) and race/ethnicity (Hispanic vs non-Hispanic) (Model 2). The presumed lower risk categories [e.g., HER−, ER+, PR+, lower stage (0/I), lower grade (I/II), later age at menarche (≥ 12 years), parous, physically active, lower BMI (< 25 kg/m^2^), and lower TBF (≤ 46%)] were used as the reference groups in the NBR analysis. The mean estimate ratio (MER) under the NBR model represents the ratio of the log estimate in one group versus the reference group and the *p* value is the probability of obtaining such a ratio under the null hypothesis. Thus, if the mean abundance of a taxon is higher in the HER2+ than in the HER2− group (reference group), we expect a MER greater than one. On the other hand, if the mean abundance of a taxon is lower among HER2+ than HER2− tumors, we expect a MER less than one. A probability of *P* ≤ 0.001 was accepted as significant in this study. Results were similar for Model 1 and 2 and we showed statistically significant MERs in NBR from Model 2 (Tables 3, 4, 5 and 6). For this pilot study we did not adjust for multiple testing [22]. All data were analyzed using R (R Foundation for Statistical Computing Vienna, Austria or SAS version 9.4 (SAS, Cary, NC).

## Results

The 37 breast cancer patients had an average age of 50.6 ± 12.3, 73% were Hispanic (*n* = 27), 54% were premenopausal (*n* = 20), 21% (*n* = 8) were nulliparous, mean age of menarche of 12.4 ± 1.5, and baseline BMI of 30.6 ± 7.9 kg/m^2^ and TBF of 42.7% ± 6.9. Most had early stage (I/II) (*n* = 22, 59.5%), high grade (III) (*n* = 23, 62.2%), hormone receptor positive (ER+PR+) (*n* = 23, 62.2%), and HER2− breast cancer (*n* = 25, 67.6%) (Table 1). Women with HER2+ breast cancer were more likely to have PR− breast cancer; 66.7% of patients with HER2+ breast cancer had PR− breast cancer compared to 24% of those with HER2− breast cancer (*p* = 0.001).

### PERMANOVA analysis of personal and tumor characteristics with the unweighted UniFrac distance matrix

**Fig. 2:**
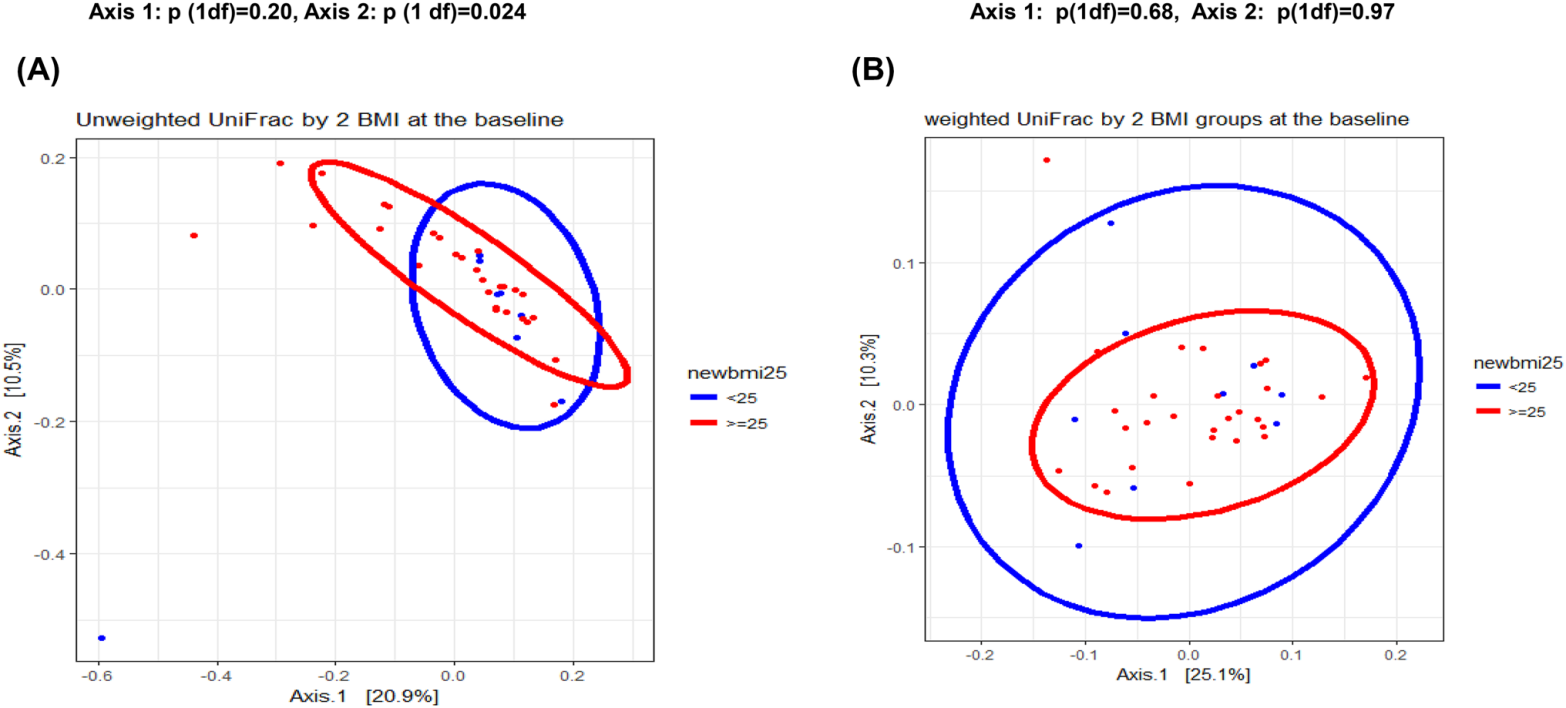
Beta-diversity results by baseline body mass index are shown: **A** unweighted UniFrac-based principal component analysis plot of the first two principal coordinates categorized by body mass index (BMI < 25 kg/m^2^*n* = 9, BMI ≥ 25 kg/m^2^*n* = 28). Axis 1 explained 20.9% while axis 2 explained 10.5% of the variance. **B** Weighted UniFrac-based principal component of the first two principal coordinates categorized by BMI; axis 1 explained 25.1% and axis 2 explained 10.3% of the variance

Beta diversity (between-subjects species diversity) was assessed using the unweighted and weighted UniFrac distance. BMI was associated with baseline gut microbiome composition. Axis 1 explained 20.9% of all variance while axis 2 explained 10.5% (Fig. 2). Separation between the baseline microbiota of the BMI groups (< 25 vs ≥ 25 kg/m^2^) differed for axis 1 (*p* = 0.20) and axis 2 (*p* = 0.024) with the unweighted UniFrac distance matrix but not with the weighted UniFrac distance (Fig. 2). Separation of baseline microbiota was also observed using cutpoints of < 30 vs ≥ 30 for BMI (axis 1 *p* = 0.16; axis 2 *p* = 0.009) and < 46% vs ≥ 46% for TBF (axis 1 *p* = 0.21; axis 2 *p* = 0.048). None of the other factors were associated with overall fecal composition (data not shown).

### Alpha diversity by tumor characteristics and personal characteristics

There were no statistically significant baseline alpha diversity (within-subject species diversity) differences by tumor stage and grade, ER or PR status (Table 2). However, alpha diversity measures were 12% to 23% lower for HER2+ (*n* = 12) than HER2− (*n* = 25) breast cancer; including lower OTU (*p* = 0.033), Chao1 index (*p* = 0.073), and Shannon index (*p* = 0.035). High (> 46%) TBF compared to lower (≤ 46%) TBF was associated with lower Chao 1 index (*p* = 0.011) and OTU (*p* = 0.059). Similar patterns of differences were observed for those with normal BMI versus overweight or obese. Alpha diversity measures were lower among women with early (≤ 11) than later (≥ 12) age of menarche; these differences were statistically significant for OTU (*p* = 0.034), Chao 1 index (*p* = 0.020) and borderline statistically significant for Shannon index (*p* = 0.057) and PD whole tree (*p* = 0.073). Those who were physically active had higher Chao 1 index (*p* = 0.07) and OTU  (*p* = 0.58) than those who were not physically active but Shannon index and PD tree were not higher. Alpha diversity measures did not differ between parous and nulliparous women.

**Table 2:** Median baseline alpha diversity measures^a^ by select tumor characteristics and breast cancer risk factors

	*N*	Observed species	Chao1	Shannon	PD tree
Age					
< 50	20	37.50	69.05	3.07	12.65
50+	17	35.00	66.00	3.00	11.97
* p* value		0.39	0.43	0.86	0.17
Stage					
I/II	22	34.00	56.06	2.98	11.79
III	15	35.00	68.50	2.98	11.97
* p* value^b^		0.84	0.80	0.38	0.65
Grade					
I/II	14	31.00	54.00	2.97	11.46
III	23	36.50	67.33	2.99	12.30
* p* value^b^		0.25	0.40	0.46	0.33
ER status					
Positive	28	33.50	60.86	2.97	11.72
Negative	9	36.00	66.00	2.99	12.62
* p* value^b^		0.64	0.87	0.53	0.36
PR status					
Positive	23	33.50	57.49	2.97	11.72
Negative	14	36.00	68.50	2.99	12.62
* p* value^b^		0.93	0.93	0.93	0.62
HER2 status					
Positive	12	26.00	53.00	2.71	10.88
Negative	25	36.50	69.17	3.07	12.42
* p* value^b^		0.033	0.073	0.035	0.11
BMI (kg/m^2^)					
< 25	9	38.00	71.58	3.12	12.49
≥ 25	28	33.00	58.13	2.92	11.97
* p* value^b^		0.091	0.24	0.11	0.33
Total body fat (TBF)					
≤ 46%	25	36.52	72.41	3.03	12.11
> 46%	12	31.17	49.99	2.91	11.05
* p* value^b^		0.059	0.011	0.35	0.26
BMI &TBF					
I (< 25 & ≤ 46)	9	38.56	74.67	3.16	12.34
II (≥ 25 & ≤ 46)	16	35.38	71.13	2.96	11.97
III (≥ 25 & > 46)	12	31.17	49.99	2.91	11.05
*p* (2df)		0.11	0.038	0.38	0.50
Age menarche					
≤ 11	11	29.18	50.51	2.73	10.25
≥ 12	26	35.38	69.74	3.00	11.89
* p* value^b^		0.034	0.020	0.057	0.073
Livebirths					
None	8	35.5	51.8	3.04	11.72
1+	29	33.0	66.0	2.89	12.11
* p* value^b^		0.81	0.77	0.91	0.71
Physical activity^c^					
No	13	34.00	48.75	3.11	12.53
Yes	24	37.00	69.17	3.00	12.30
* p* value^b^		0.58	0.07	0.82	0.31

^a^Rarefaction of 100

^b^*p* obtained by Wilcoxon rank sum test

^c^No strenuous, vigorous or moderate activity per week

### Phyla abundance differences by tumor characteristics and breast cancer risk factors

**Fig. 3:**
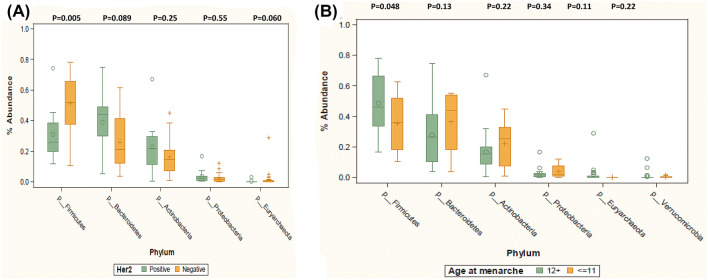
Relative abundance levels of the most frequent phyla among **A** breast cancer patients with HER2+ tumors (*n* = 12) vs HER2− tumors (*n* = 25), and **B** breast cancer patients with early age at menarche (≤ 11) (*n* = 11) vs later age at menarche (≥ 12) (*n* = 26) are shown. Wilcoxon rank sum test was used to test for phylum-level differences by HER2 status and by age at menarche. *p* values are listed above each phylum

**Fig. 4:**
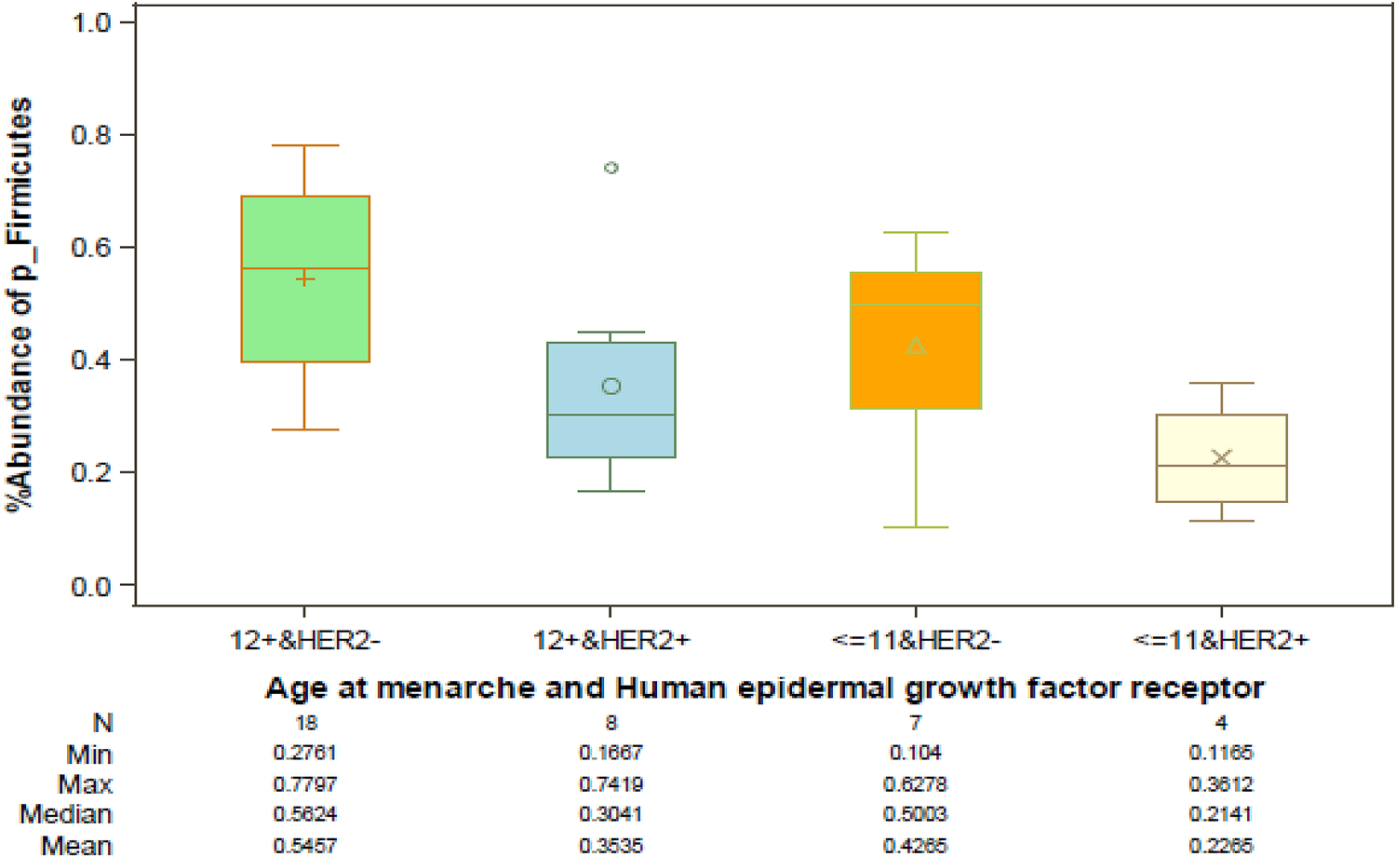
Relative abundance levels (mean, median, minimum and maximum) of *Firmicutes* by four groups of breast cancer patients are shown: HER2− breast cancer and later age at menarche (≥ 12) (*n* = 18), HER2+ breast cancer and late age at menarche (*n* = 8), HER− breast cancer and early age at menarche (≤ 11) (*n* = 7), and HER2+ breast cancer and early age at menarche (*n* = 4)

There were no significant phyla differences by ER and PR status, stage, grade, parity, BMI, and TBF% (data not shown). However, median level of *Firmicutes* was lower among women with HER2+ than those with HER2− breast cancer (33.53 vs 51.75, *p* = 0.005*),* and also lower among women with early (≤ 11) than those with later (≥ 12) age of menarche (35.61 vs 50.17, *p* = 0.048) (Fig. 3). We explored differences in abundance by age at menarche and HER2 status combined (Fig. 4). Levels of Firmicutes were highest among those who had HER2− and menarche age ≥ 12 (56.24%), intermediate among those who had HER2− and menarche age ≤ 11 (50.03%) or HER2+ and menarche age ≥ 12 (30.4%), and lowest among those with HER2+ and menarche age ≤ 11 (21.4%) (p_3df_ = 0.009). These results suggest an association of HER2 status with levels of *Firmicutes* among those with age at menarche at ≥ 12 (*p* = 0.027), and a borderline association of age at menarche with *Firmicutes* among women with HER2− breast cancer (*p* = 0.105). The largest difference was between those who differed by both HER2 status and age at menarche (56.24% vs 21.4%, *p* = 0.006).

### Taxa abundance differences by ER, PR, and HER2 status

**Fig. 5:**
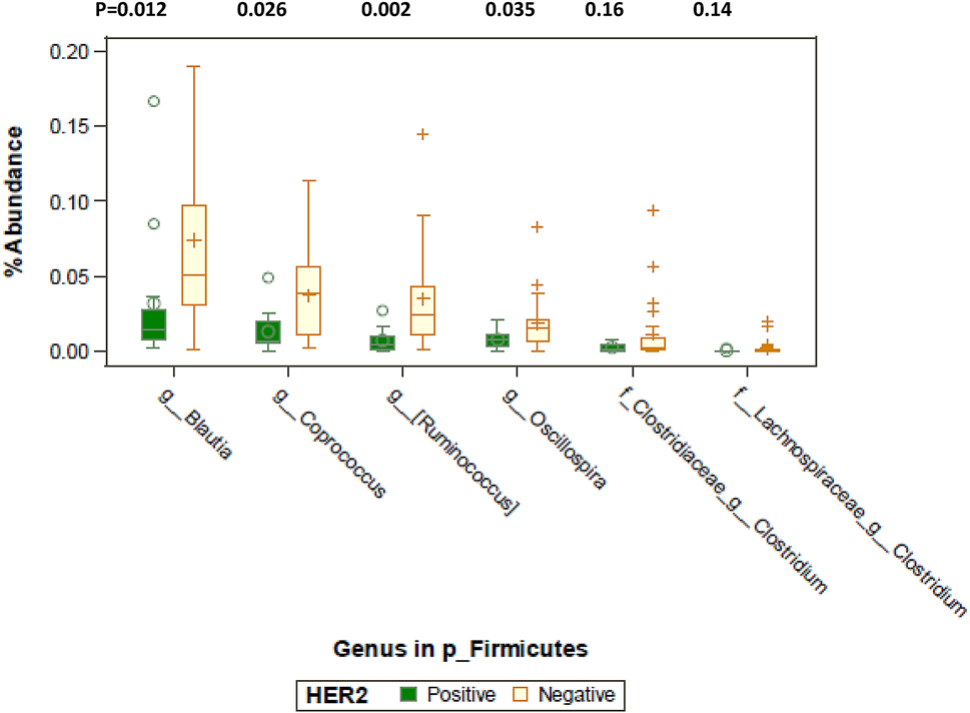
Relative abundance levels of select genera of *Firmicutes* by HER2 status are shown. Wilcoxon rank sum test was used to test for genus-level differences by HER2 status. *p* values are listed above each genus

Table 3 results showed MERs that differed significantly by ER, PR and HER2 status after adjusting for total counts, age, and race/ethnicity. MER > 1 denotes higher taxa abundances in ER− than ER+, PR− than PR+, and HER2+ than HER2− breast cancers whereas MER < 1 shows lower taxa abundances in ER− than ER+, PR− than PR+, and HER2+ than HER2− breast cancers. In total, 13 taxa differed between those with HER2+ vs HER2− tumors (*p* ≤ 0.001), 3 taxa differed between ER+ and ER− tumors, and 2 taxa differed between PR+ and PR− tumors. The taxa that differed between HER2+ vs HER2− tumors included specific *Bacteroidetes (g_Alistipes), Firmicutes (g_Enterococcus, g_Acidaminococcus)* showing higher abundances (MER > 1) in HER2+ than HER2−. Other *Bacteroidetes (f_Rikenellaceae), Euryarchaeto (g_Methanobrevibacter), Firmicutes (f_Christensenellaceae, g_Turicibacter, g_Clostridium, g_SMB53, g_Blautia, g_Coprococcus, g_Ruminococcus*), and *Proteobacteria (g_Desulfovibrio*) showed lower abundances in HER2+ than HER2− tumors. Abundance of three *Firmicutes* taxa (*g_Enterococcus*, *g_Turicibacter, g_Veillonella)* and one *Proteobacteria taxa (g_Haemophilus)* were lower in ER+ than ER−. Three *Firmicutes* taxa (*g_Turicibacter, f_Clostridiaceae:g_Clostridium, f_Erysipelotrichaceae:g_Clostridium)* were lower in PR+ than PR− breast cancers. The unadjusted relative abundances of select *Firmicutes* by HER2 status are displayed in Fig. 5, in support of the results shown by MER in Table 3.

**Table 3:** Mean ratio estimates (MER)^a^ obtained by zero-inflated negative binomial model of taxa abundances by estrogen receptor (ER), progesterone receptor (PR), and human epidermal growth factor receptor 2 (HER2) status with adjustment for total counts, age and race/ethnicity (model 2, MER)

			ER− vs ER+		PR− vs PR+		HER2+ vs HER2−	
			MER	*p* value	MER	*p* value	MER	*p* value
p__Bacteroidetes								
	f__Rikenellaceae						.039	.0060
	f__Rikenellaceae	g__Alistipes					4.953	.0075
p__Euryarchaeota								
	f_Methanobacteriaceae	g_Methanobrevibacter					.001	.0039
p__Firmicutes								
	f__Enterococcaceae	g__Enterococcus	.045	.0037			59.538	.0012
	f__Turicibacteraceae	g__Turicibacter	.034	.0092	.114	.0031	.157	.0050
	f__Chistenseneitaceae	g_					.085	.0002
	f__Clostridiaceae	g__Clostridium			.184	.0015	.165	.0023
	f__Clostridiaceae	g__SMB53					.214	.0046
	f__Lachnospiraceae	g__Blautia					.409	.0085
	f__Lachnospiraceae	g__Coprococcus					.405	.0077
	f__Lachnospiraceae	g__[Ruminococcus]					.287	.0002
	f__Veillonellaceae	g__Acidaminococcus					244.94	.0003
	f__Veillonellaceae	g__Veillonella	.074	.0003				
	f__Erysipelotrichaceae	g__Clostridium			.058	.0051		
p__Proteobacteria								
	f__Desulfovibrionaceae	g__Desulfovibrio					.059	.0005
	f__Pasteurellaceae	g_Haemophilus	.014	< .0001				

^a^MER > 1 means higher taxa in ER− than ER+, PR− than PR+, and HER2+ than HER2− group; ER+, PR+, and HER2− was the respective reference group

### Taxa abundance differences by stage and grade

Two taxa of *Firmicutes (g_Clostridium, g_Veillonella)* were more abundant (MER > 1) among women with higher grade (III) or higher stage breast cancers compared to lower grade (I/II) or lower stage breast cancers. In addition, higher grade was associated with higher abundance of *Actinobacteria (g_Eggerthella)* but lower abundance (MER < 1) of other taxa of *Actinobacteria (f_Coriobacteriaceae*), and *Firmucutes (f_Lachnospiraceae, g_Anaerostipes, f_Ruminococcaceae)* (Table 4). Higher stage breast cancer was also associated with higher abundance of *Firmicutes (f_Clostridiaceae)* and *Proteobacteria (f_Enterobacteriaceae, g_Haemophilus)* but lower abundance of *Firmicutes (g_Acidaminococcus, g_Catenbacterium)* (Table 4).

**Table 4:** Mean estimate ratios (MER)^a^ obtained by zero-inflated negative binomial model of taxa abundances by grade and  stage of breast cancer with adjustment for total counts, age and race/ethnicity (model 2, MER)

			Grade high (III) vs low (I/II)		Stage high (III) vs low (I/II)	
			MER	*p* value	MER	*p* value
p__Actinobacteria						
	f__Coriobacteriaceae	g__	.238	0.0028		
	f__Coriobacteriaceae	g__Eggerthella	9.365	0.0004		
p__Firmicutes						
	f__Clostridiaceae	g__			3.290	.0011
	f__Clostridiaceae	g__Clostridium	6.144	0.0088	5.986	.0005
	f__Lachnospiraceae		.343	0.0003		
	f__Lachnospiraceae	g__Anaerostipes	.116	< 0.0001		
	f__Ruminococcaceae		.488	0.0066		
	f__Veillonellaceae	g__Acidaminococcus			.0098	.0003
	f__Veillonellaceae	g__Veillonella	9.794	0.0025	15.12	< .0001
	f__Erysipelotrichaceae	g__Catenibacterium			.151	.0002
p__Proteobacteria						
	f__Enterobacteriaceae				6.389	.0024
	f__Pasteurellaceae	g_Haemophilus			71.633	< .0001

^a^MER > 1 means higher taxa in high grade (III) than low grade (I and II) and in high stage (III) than low stage (I and II); low grade and low stage was the respective reference group

### Taxa abundance differences and breast cancer risk factors

We also explored whether there are taxa differences by treating older age at diagnosis (≥ 50 years), later age at menarche, parous, BMI (< 25 kg/m^2^), TBF (≤ 46%), and physically active as the reference groups in the NBR model analysis (Tables 5 and 6). Younger women at diagnosis (< 50 years) (higher risk) compared to older age at diagnosis displayed higher abundance (MER > 1) in five taxa including *Actinobacteria (g_Eggerthella) and Firmicutes (f_Clostridiaceae, g_SMB53, g_Clostridium, g_Lactococcus).* Women who reported menarche age ≤ 11 (higher risk) compared to ≥ 12 menarche age showed significant differences in nine taxa, including lower abundance (MER < 1) of *Actinobacteria (f_Coriobacteriaceae), Euryarchaeota (g_Methanobrevibacter)* and *Firmicutes (g_Turicibacter, g_Anaerostipes, g_Lachnobacterium, f_Ruminococcaceae, g_Ruminococcus)* but higher abundance (MER > 1) of *Firmicutes (f_Lachnospiracaceae:g_Clostridium) and Proteobacteria (g_Escherichla).* Nulliparous compared with parous women displayed lower abundance (MER < 1) of two genera of *Firmicutes (g_Lactococcus, g_Catenibacterium*) but higher abundance (MER > 1) of *Actinobacteria (g_Actinomyces) and Proteobacteria (g_Bilophila)*.

**Table 5:** Mean estimate ratios (MER)^a^ obtained by zero-inflated negative binomial model of taxa abundances by age group^b^, menarche age and parity^c^

			Age (≤ 50 vs 50+)^b^		Menarche Age^c^ ≤ 11 vs ≥ 12		Nulliparous vs Parous^c^	
			MER	*p* value	MER	*p* value	MER	*p* value
p__Actinobacteria								
	f__Actinomycetaceae	g__Actinomyces					4.006	.0068
	f__Coriobacteriaceae	g__Eggerthella	6.0133	0.002				
	f__Coriobacteriaceae	g__			.2447	.0062		
p__Bacteroidetes								
	f__Methanobacteriaceae	g__Methanobrevibacter			.0081	.001		
p__Firmicutes								
	f__Clostridiaceae		12.5643	< .0001				
	f__Clostridiaceae	g__SMB53	7.2232	0.0068				
	f__Erysipelotrichaceae	g__Catenibacterium					.0104	.0001
	f__Erysipelotrichaceae	g__Clostridium	19.9947	< .0001				
	f__Lachnospiraceae	g__Anaerostipes			.0403	.0029		
	f__Lachnospiraceae	g__Clostridium			8.280	.0086		
	f__Lachnospiraceae	g__Lachnobacterium			.0143	.0011		
	f__Streptococcaceae	g__Lactococcus	32.8322	< .0001			.0419	.0055
	f__Turicibacteraceae	g__Turicibacter			.0874	.0028		
	f__Ruminococcaceae				.4229	.0016		
	f__Ruminococcaceae	g__Ruminococcus			.2068	.0004		
P_Proteobacteria								
	f__Enterobacteriaceae		10.4271	0.0005				
	f__Desulfovibrionaceae	g__Bilophila					3.0562	.0064
	f__Enterobacteriaceae	g__Escherichla			31.523	< 0.0001		

^a^MER > means higher taxa in women aged < 50, early menarche age (≤ 11), nulliparous, high BMI (≥ 25), high TBF(> 46%) than age 50+, later menarche (≥ 12), parous, low BMI, and low TBF, respectively

^b^Adjustment for total counts and race/ethnicity

^c^Adjustment for total counts, age and race/ethnicity in analysis on age at menarche and parity (model 2, MER)

**Table 6:** Mean estimate ratios (MER)^a^ obtained by zero-inflated negative binomial model of taxa abundances by BMI, total body fat, and physical activity with adjustment for total counts, age and race/ethnicity (model 2, MER)

			BMI (kg/m^2^) ≥ 25 vs < 25		Total body fat (TBF) > 46% vs ≤ 46%		Physical activity (none vs yes)	
			MER	*p* value	MER	*p* value	MER	*p* value
p__Actinobacteria								
	f__Coriobacteriaceae	g__			.0661	< .0001	0.1418	0.0004
p__Firmicutes								
	f__Lactobacillaceae	g__Lactobacillus	.053	.0083				
	f__Streptococcaceae	g_Streptococcus	.134	.0012				
	f__Clostridiaceae		1.985	.0074	7.909	< .0001	0.1023	< .0001
	f__Clostridiaceae	g__Clostridium			6.901	.0033		
	f__Lachnospiraceae	g__Lachnobacterium					0.0275	0.0004
	f__Lactobacillaceae	g__Lactobacillus					0.0388	0.0015
	f__Lachnospiraceae	g__Lachnospira			3.127	.0085		
	f__Veillonellaceae	g__Veillonella					12.3926	0.0007
	f__Erysipelotrichaceae	g__Catenibacterium			.0809	.002		
p__Verrucomicrobia								
	f__Verrucomicrobiaceae	g__Akkermansia	181.63	< 0.0001				

^a^MER > means higher taxa in high BMI (≥ 25), high TBF(> 46%), and no regular physical activity than low BMI, and low TBF and yes regular physical activity

Differences in select taxa emerged in comparisons by BMI (< 25 vs ≥ 25 kg/m^2^) and TBF (< 46% vs ≥ 46%); BMI and TBF were highly correlated (R^2^ = 0.61, *p* < 0.0001) (Table 6). Women with BMI ≥ 25 kg/m^2^ compared to those with lower BMI displayed higher abundance (MER > 1) of *Firmicutes (f_Clostridiaceae)* and V*errucomicrobia (g_Akkermansia)* but lower abundance (MER < 1) of *Firmicutes (g_Lactobacillus, g_Streptococcus).* When we examined difference in taxa by TBF, women with higher TBF (≥ 46%) compared to those with lower TBF (< 46%) also showed higher abundance (MER > 1) of *Firmicutes (f_Clostridiaceae*, g*_Clostridium, g_Lachnospira*) but lower abundance (MER < 1) of *Actinobacteria (f_Coriobacteriaceae) and Firmiciutes (g_Catenbacterium).* There are some taxa differences between those who were physically active compared to those who were inactive; including lower abundance of some *Firmicutes (f_Clostridiaceae; g_Lachnobacterium, g_Lactobacillus)* but higher abundance of other *Firmicutes (f_ Veillonella).*

## Discussion

We investigated the gut microbiome profile in relation to ER/PR and HER2 status, tumor grade and stage, and select breast cancer risk factors in 37 women diagnosed with incident breast cancer; most of whom (73%) were Hispanics, and were overweight or obese (75%). Women with HER2+ compared with HER2− breast cancers displayed a less diverse microbiome and a distinct bacterial composition profile, including in abundance of *Firmicutes* (see below). Breast cancer patients with high (≥ 46%) TBF and earlier age at menarche (≤ 11) also had a less diverse gut microbiome. Abundance of Firmicutes was significantly lower among women with HER2+ breast cancer and early menarche than those with HER2− breast cancer and later menarche. Before we interpret these new results, we discuss our results on body size comparisons and tumor grade and stage in relation to published findings.

Alpha diversity measures have been used as a hallmark of health habits including adherence to Mediterranean diets [23–25] and body composition [26]. Lower gut alpha diversity has been associated with human obesity in a meta-analysis, showing significant relationships between obesity and microbial richness, evenness, and diversity [26]. Chao 1 index and OTU were 31% (*p* = 0.011) and 14% (*p* = 0.059) lower among women with > 46% TBF compared to those with ≤ 46% TBF; similar but weaker patterns were observed by BMI (Table 2). Associations between various bacterial groups and BMI have been reported but a consistent taxonomic signature of obesity has not been identified [27, 28]. Women in this study with higher BMI or higher TBF displayed higher abundance of *Firmicutes (f_Clostridiaceae).* Additionally, those with higher BMI displayed higher abundance of *g_Akkermansia;* enrichment of this taxa has been related with body composition in other studies [29–31]. Several sub-taxa within *Firmicutes (g_Streptococcus*) associated with lower BMI [28, 31, 32] also appeared to differ by BMI in this study. However, small numbers of those with BMI < 25 kg/m^2^ (*n* = 9) may have limited our ability to identify other taxa that have been associated with lean/normal BMI (e.g.,* f_Christensenellaceae; g_Oscillospira*) [23, 33, 34]. Interestingly, breast cancer patients without regular physical activity also showed lower Chao 1 index (*p* = 0.07) and tended to have lower abundance of several taxa of *Firmicutes (f_Clostridiaceae)* in support of growing evidence that exercise favorably influences the function and composition of human gut microbiota] [35] However, limited sample size precluded our ability to examine the combined effects of physical activity and finer categories of BMI on microbiome diversity and composition. Results from a large study showed that microbiome differences by BMI may be missed if categories of BMI comparisons are crude. In this previous study, microbiome composition did not differ between normal weight (< 25 kg/m^2^) and overweight (25–30 kg/m^2^) persons, but there were significant differences in microbiome between normal weight and those who had class I obesity (> 30–≤ 35) or class II obesity > 35 kg/m^2^ [28].

Our findings on taxa differences by breast cancer grade and stage add to results from one previous study of mostly low grade (77% were grade I/II) and low stage (59% stage 0/I) breast cancers [7]. A higher abundance of *g_Clostridium* was found among those with higher tumor grade or stage in this study, similar to the finding of abundance of *Clostridium coccoides cluster* in the previous study [7]. Moreover, women with higher grade or higher stage breast cancers also displayed higher abundance of *f_Veillonella* but lower abundance of *f_Erysipelotrichaeceae* which has been related with inflammation-related conditions [36]. The significance of our finding of high abundance of taxa in *p_Proteobacteria (g_Haemophilus, f_Enterobacteriaceae*) among those with higher tumor stage is not clear but it is intriguing that *g_Haemophilus* appeared to be over-represented among individuals with impaired glucose regulation [36].

Reasons for the lower alpha diversity among women with HER2+ compared to those with HER2− breast cancer are not known. Menarche age, parity, BMI, and TBF did not differ by HER2 status. It is intriguing that women with HER2+ compared to those with HER2− breast cancer displayed lower abundance of select genera of *Firmicutes *(e.g.,* g_Clostridium, g_Blautia, g_Coprococcus, g_Ruminococcus, g_SMB53*) and higher abundance of select genera of *p_Bacteroidetes*; thus a deficit of taxa that have often been linked with healthy body composition, body leanness and healthy metabolic profile [37, 38]. Lower weight gain has been associated with taxa of the *Ruminococcaceae* family in studies of twins [27].

Another novel finding is that earlier menarche age was associated with lower alpha diversity; these findings were statistically significant for OTU and Chao1 index. Age at menarche is likely a marker of earlier life diet and nutrition [39].  Earlier age at menarche has been found to have a lasting effect [40], conferring higher circulating estradiol levels for those who started to menstruate at ages 11 or younger than at age 14 or older (*p* = 0.033) [41]. High gut microbial diversity has been associated with a profile of estrogen metabolites associated with reduced breast cancer risk [42]. Levels of urinary estrogen metabolites have been correlated with relative abundances of specific *Clostridia* taxa [42, 43]. There are likely bidirectional influences between sex steroids and the gut microbiome. Various bacterial genes have been found to affect β-glucuronidase enzymatic activity, influencing deconjugation and reabsorption of estrogens. Levels of circulating estrogen, in turn, may influence the abundance of certain bacteria species [42–47].

Strengths of this pilot study include our collection of detailed information on relevant breast cancer risk factors and tumor characteristics and considering them in this analysis using two complementary methods, by Wilcoxon rank sum test and a zero-inflated NBR model with adjustment for select covariates. This study included mostly Hispanics in the catchment area of USC. However, we are limited by our cross-sectional analyses and modest sample size so that we used only two categories in our comparisons of taxa differences by age at menarche, parity, physical activity, BMI and TBF%. Breastfeeding, a parity-related factor, that has emerged as an important modifiable lifestyle factor for breast cancer, was not asked in our study. Research regarding the association of specific microbiome taxa to disease or other conditions inherently involves studying the relationships of numerous taxa with multiple conditions, thus greatly increasing the possibility of type 1 errors. On the other hand, small sample sizes preclude the recognition of any but the strongest associations when very small alpha-levels are used for statistical significance. Even with our conservative *α*-level of 0.001 we found far more statistically significant results than would be expected by chance alone, particularly with respect to HER2, grade, and age at menarche. Although some of these findings may be chance findings, while other important associations may have been missed due to the small alpha used, we feel that we have struck a reasonable balance, and that these findings are informative and warrant further consideration.

## Conclusions

In summary, this pilot cross-sectional study of mostly Hispanic women found that HER2 status and age at menarche had significant associations with gut microbiome alpha diversity measures and specific microbial composition. These findings warrant confirmation in studies with larger sample sizes of diverse racial/ethnic groups and with repeated sample collections to determine how microbiome are associated with breast cancer subtypes and specific risk factors.

## Abbreviations

BMI: Body mass index
DEXA: Dual-energy X-ray absorptiometry
ER/PR: Estrogen/progesterone receptor
HR: Hormone receptor
HER2: Human epidermal growth factor receptor 2
MER: Mean estimate ratio
NBR: Negative binomial regression
OTU: Number of species
PERMANOVA: Permutational multivariate analysis of variance
PD: Phylogenetic distance
PCoA: Principal coordinate analysis
rRNA: Ribosomal RNA
TBF: Total body fat
USC: University of Southern California
